# Public Adoption of and Trust in the NHS COVID-19 Contact Tracing App in the United Kingdom: Quantitative Online Survey Study

**DOI:** 10.2196/29085

**Published:** 2021-09-17

**Authors:** Liz Dowthwaite, Joel Fischer, Elvira Perez Vallejos, Virginia Portillo, Elena Nichele, Murray Goulden, Derek McAuley

**Affiliations:** 1 Horizon Digital Economy Research University of Nottingham Nottingham United Kingdom; 2 School of Computer Science University of Nottingham Nottingham United Kingdom; 3 National Intitute for Health Research Biomedical Research Centre Institute of Mental Health, Division of Psychiatry and Applied Psychology University of Nottingham Nottingham United Kingdom; 4 School of Sociology and Social Policy University of Nottingham Nottingham United Kingdom

**Keywords:** trust, technology adoption, COVID-19, digital contact tracing, coronavirus, vulnerable populations, attitudes, SARS-CoV-2, digital proximity tracing, compliance

## Abstract

**Background:**

Digital contact tracing is employed to monitor and manage the spread of COVID-19. However, to be effective the system must be adopted by a substantial proportion of the population. Studies of mostly hypothetical contact tracing apps show generally high acceptance, but little is known about the drivers and barriers to adoption of deployed systems.

**Objective:**

The aim of this study was to investigate adoption of and attitudes toward the NHS (National Health Service) COVID-19 smartphone app, the digital contact tracing solution in the United Kingdom.

**Methods:**

An online survey based on the extended Technology Acceptance Model with the added factor of trust was carried out with a representative sample of the UK population. Statistical analysis showed adoption rates, attitudes toward and trust in the app, and compliance with self-isolation advice and highlighted differences for vulnerable populations (ie, older adults aged 65 years and over and members of Black, Asian, and minority ethnic [BAME] communities).

**Results:**

A total of 1001 participants took part in the study. Around half of the participants who had heard of the NHS COVID-19 mobile phone app (490/963, 50.9%; 95% CI 47.8%-54.0%) had downloaded and kept the app, but more than one-third (345/963, 35.8%; 95% CI 32.8%-38.8%) either did not intend to download it or had deleted it. Significantly more BAME respondents than White respondents had deleted the app (16/115, 13.9%; 95% CI 11.8%-16.0%, vs 65/876, 7.4%; 95% CI 5.8%-9.0%), and significantly more older adults 65 years and over than those under 65 years did not intend to download it (44/127, 34.6%; 95% CI 31.7%-37.5%, vs 220/874, 25.2%; 95% CI 22.5%-27.9%). Broadly, one of the reasons for uptake was to help the NHS and other people, especially among older adults, although significantly fewer BAME participants agreed that they did so to help the NHS. Reported compliance with received notifications to self-isolate was high but was significantly lower than reported intended compliance without received notifications. Only one-fifth (136/699, 19.5%; 95% CI 17.0%-22.0%) of participants understood that the decision to send self-isolation notifications was automated by the app. There were a range of significantly more negative views among BAME participants, including lower trust in the NHS, while older adults were often significantly more positive. Respondents without the app reported significantly lower trust and more negative views toward the app and were less likely to report that they understood how the app works.

**Conclusions:**

While compliance on the part of the approximately 50% of participants who had the app was fairly high, there were issues surrounding trust and understanding that hindered adoption and, therefore, the effectiveness of digital contact tracing, particularly among BAME communities. This study highlights that more needs to be done to improve adoption among groups who are more vulnerable to the effects of the virus in order to enhance uptake and acceptance of contact tracing apps.

## Introduction

Digital contact tracing solutions are employed to monitor and manage the spread of disease during pandemics [1]. Public acceptance of app-based contact tracing in the United Kingdom, the European Union, and the United States is high [2-4]; however, to make a difference they must be adopted by a substantial proportion of the population [5,6]. Engaging users in the development, implementation, and evaluation of contact tracing can help maximize engagement and technology acceptance, according to the Responsible Research and Innovation (RRI) framework [7,8]. Therefore, this paper reports on research to understand the drivers and barriers to adoption of a COVID-19 contact tracing app in the United Kingdom to help increase the effectiveness of such systems and inform future design.

An earlier study of hypothetical digital contact tracing in the United Kingdom suggested that people would adopt it to protect family, friends, and the community as well as to stop the pandemic, while potential barriers were reported risks of postpandemic surveillance, increasing anxiety, and fear of hacking [2,4]. In a study by Velicia-Martin et al, the Technology Acceptance Model (TAM) was used to show that the intention to use a contact tracing app was determined by how useful it was perceived to be; the study also showed that concern about privacy would be overridden by concerns about health [9]. Similarly, a study in Germany looked at the difference between the perceived utility of a contact tracing app and a data donation app. Motivations for using and accepting a contact tracing app were higher, and the data donation app was perceived as having less utility for the user [10]. However, there may also be wider social implications, such as having no choice but to download the app for work or venue check-ins, and real-world uptake might differ from usage within a trial.

Trust may also significantly impact the adoption of contact tracing apps, [11]; for example, a study conducted in Switzerland suggested that higher levels of trust in government and health authorities may also lead to increased uptake [12]—perceived effectiveness of a contact tracing app and the overall app user experience depended on the app being embedded within the health system. In the UK context, this may mean that trust or confidence in the National Health Service (NHS) might influence people’s attitudes toward and usage of the app. In Germany, it was also shown that general trust in official app providers as well as social trust played important roles, highlighting the importance of both data-securing issues and interpersonal solidarity [10]. A study across five countries—France, Germany, Italy, the United Kingdom, and the United States—also found that a lack of trust was one of the main barriers for adoption of a hypothetical contact tracing app [4].

Security and privacy have also been shown to be important. In the Netherlands, a study designed to determine the potential uptake of a contact tracing app in the Dutch population showed an adoption rate as high as 64% for apps that had a higher number of security and privacy-respecting features [13]. In Ireland, there was shown to be a high level of willingness to download a public health–backed app to augment contact tracing, with 54% of respondents definitely willing to download an app and 30% who would consider downloading it [14]. The most common reason to download the app was linked to social altruism: helping family and friends and a sense of responsibility to the wider community. The most common reason not to download the app was linked to issues of trust, privacy and data security, and fear that technology companies or the government might use the app technology for greater surveillance after the pandemic. Another study across five countries—France, Germany, Italy, the United Kingdom, and the United States—found that there was strong support for an app, whether it was subject to voluntary or automatic installation, but the study identified concerns once more about cybersecurity and privacy [4]. In Australia, 37.3% of participants in a study of 1500 citizens had downloaded the COVIDSafe app, and 27.7% refused to do so [15]. Reasons for not downloading the app included privacy and technical concerns, the belief that that app was unnecessary due to social distancing, distrust in the government, and apathy. This study also highlighted the importance of public health messages for increasing the acceptability of apps and their correct use, while also addressing concerns around privacy, data storage, and technical ability needed to operate the app; it also emphasized the importance of identifying and understanding specific barriers to the use of contact tracing apps to improve their design.

Data suggest that specific groups of the population are more at risk of dying of COVID-19, including older adults [16-18] as well as those in Black, Asian, and minority ethnic (BAME) communities [19,20]. The main challenge among the Dutch population was to increase the uptake among older adults, who were least inclined to install and use a COVID-19 contact tracing app [13]. In line with this, in Germany, age was negatively associated with the motivation for using a data donation app [10]. The risk for BAME communities, in particular, has been linked to socioeconomic factors [21,22]. Furthermore, recent studies have shown increased COVID-19 vaccine hesitancy in certain BAME communities [23-25]; however, little is known about whether hesitancy also extends to attitudes toward digital contact tracing.

At the time the study reported in this paper closed on December 21, 2020, 2,183,506 people in the United Kingdom had tested positive for COVID-19, approximately 3.2% of the population [26]. The UK government released the NHS COVID-19 app on September 24, 2020. The app is entirely automated and decentralized, and it relies on Bluetooth proximity as well as self-reporting of symptoms and test results [27,28]. The app has been downloaded more than 21 million times, suggesting a 56% uptake among the population aged 16 years and older who own a smartphone [29,30]. A recent study also showed that the app has been effective in reducing the number of positive cases of COVID-19 in the United Kingdom; 1.7 million users were contact-traced by the app, with an estimated reduction in cases during the second wave of COVID-19 by one-quarter. However, only 28% of people had actively used the app in the period leading up to this study [31]. Little is known about the views driving or hindering adoption of the app; our research addresses this gap. The study surveys public trust in and adoption of digital contact tracing in the United Kingdom, in terms of reported reasons, compliance, and understanding of the app, especially highlighting significant differences for vulnerable groups.

## Methods

### Recruitment

Ethical approval was granted for the study by the Research Ethics Committee of the authors’ institution. The recruitment was carried out by Ipsos MORI via email to a nationally representative sample, based on age, gender, and region, drawn from a randomly selected pool of participants who met the relevant criteria. There was also a 10% to 15% quota for BAME respondents, with the same process applied to ensure hitting the minimum required quota. As fieldwork progressed, they specifically targeted any quota groups that were still required to meet the final profile that was needed, again randomly selecting within those groups. Participants were incentivized for survey participation through monetary compensation paid into their panel account. As a market research agency, Ipsos MORI operates under the Market Research Society code of conduct and is General Data Protection Regulation compliant, so participants’ privacy was guaranteed. Data were only received via their survey platform in an anonymized form, so no personally identifiable information on the participants was received. A total of 2575 invitations to take part in the study were sent out.

A representative sample of 1001 members of the UK population aged 16 to 75 years took part, weighted to the known offline proportions for age within gender, region, working status, and ethnicity. Participants were asked a series of demographic questions, the full details of which are provided in Table S1 in Multimedia Appendix 1. A summary of the main self-reported characteristics is provided in Table 1.

**Table 1:** Summary characteristics of participants.

Characteristic		Participants (N=1001), n (%)
**Age (years)**		
	Under 65	874 (87.3)
	65 and over	127 (12.7)
**Gender**		
	Male	501 (50.0)
	Female	500 (50.0)
**Employment status**		
	Working	666 (66.5)
	Not working	335 (33.5)
**Education**		
	Up to General Certificate of Secondary Education (GSCE)	307 (30.7)
	Post-GSCE	694 (69.3)
**Ethnicity**		
	White	876 (87.5)
	Black, Asian, and minority ethnic	115 (11.5)
	Not stated	10 (1.0)
**Country of residence**		
	England	847 (84.6)
	Wales	48 (4.8)
	Scotland	85 (8.5)
	Northern Ireland	21 (2.1)

### Materials and Procedure

**Figure 1:**
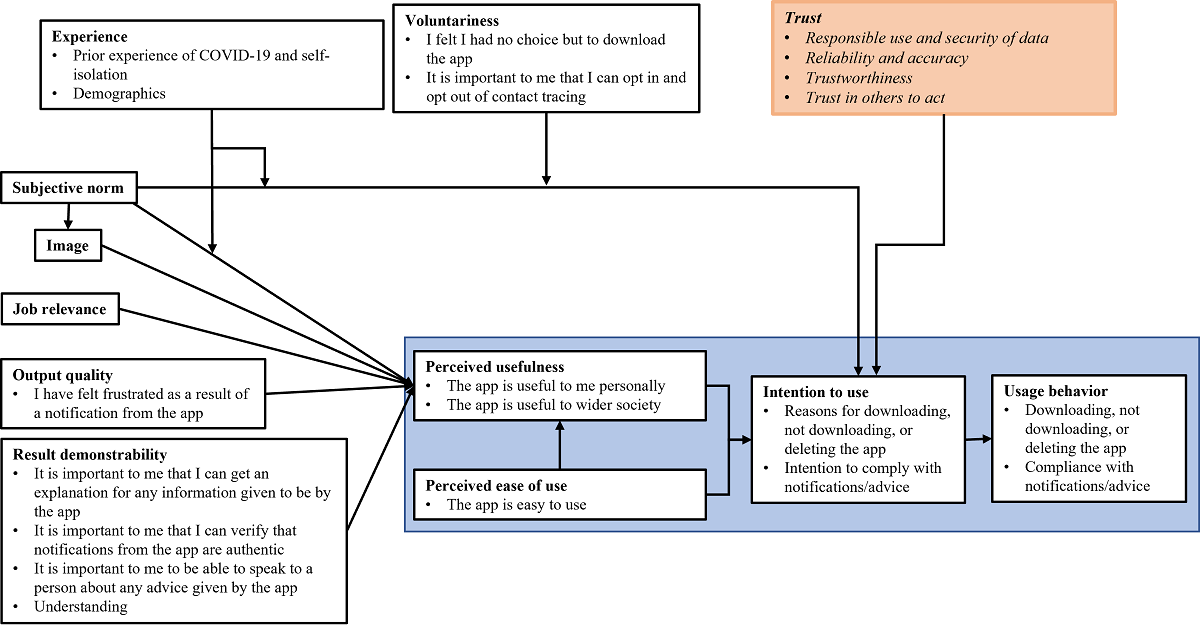
The extended Technology Acceptance Model (TAM2) and its relation to the this questionnaire study. Items in bold in white boxes are existing factors in the TAM2 [8], followed by examples, as bullet points, from the questionnaire. Trust was added as an additional factor (peach box) affecting intention to use and includes examples from the questionnaire.

An online survey was carried out between December 11 and 21, 2020, when the United Kingdom was between “lockdown 2” and “lockdown 3” and subject to a regional tier system. Questionnaire development was carried out in several stages. First, in the summer of 2020, a series of interviews were carried out with members of the public with regard to their opinions of, and intention to use, the United Kingdom’s test and trace app when it would be released (paper forthcoming). From these interviews, a series of themes were identified, which led to the survey being based on the extended TAM (TAM2) [32]. The TAM2 identifies several key factors in the adoption of new technology and has been applied to explore acceptance of various technologies, including hypothetical COVID-19 tracing apps [9]. The conceptual model is extended with “trust” as a factor for acceptance, as it may significantly impact the adoption of contact tracing apps [11]. A list of pertinent questions was developed among the author team; the questions were tested and refined involving experts in questionnaire development from Ipsos MORI. Figure 1 illustrates how these questions relate to and extend the TAM2 framework.

Recruitment and data collection were carried out by Ipsos MORI, who also carried out piloting of the questionnaire. The questionnaire had an initial data review on Day 1 with 61 respondents; it was thoroughly checked to make sure that all data were being collected correctly (eg, checking routing and displaying of correct answer options) and was checked for anomalies and understanding. The data were reviewed again when 213 respondents had completed it to ensure data quality.

Participants were provided with information and privacy notices and gave informed consent to take part. All questions were closed-ended, either multiple choice or rated on Likert or Likert-like scales from 1 (“strongly disagree” or “not at all”) to 5 (“strongly agree” or “entirely”), except for a single open-ended question that was included for further comments; participants were routed to appropriate questions based on previous answers.

**Figure 2:**
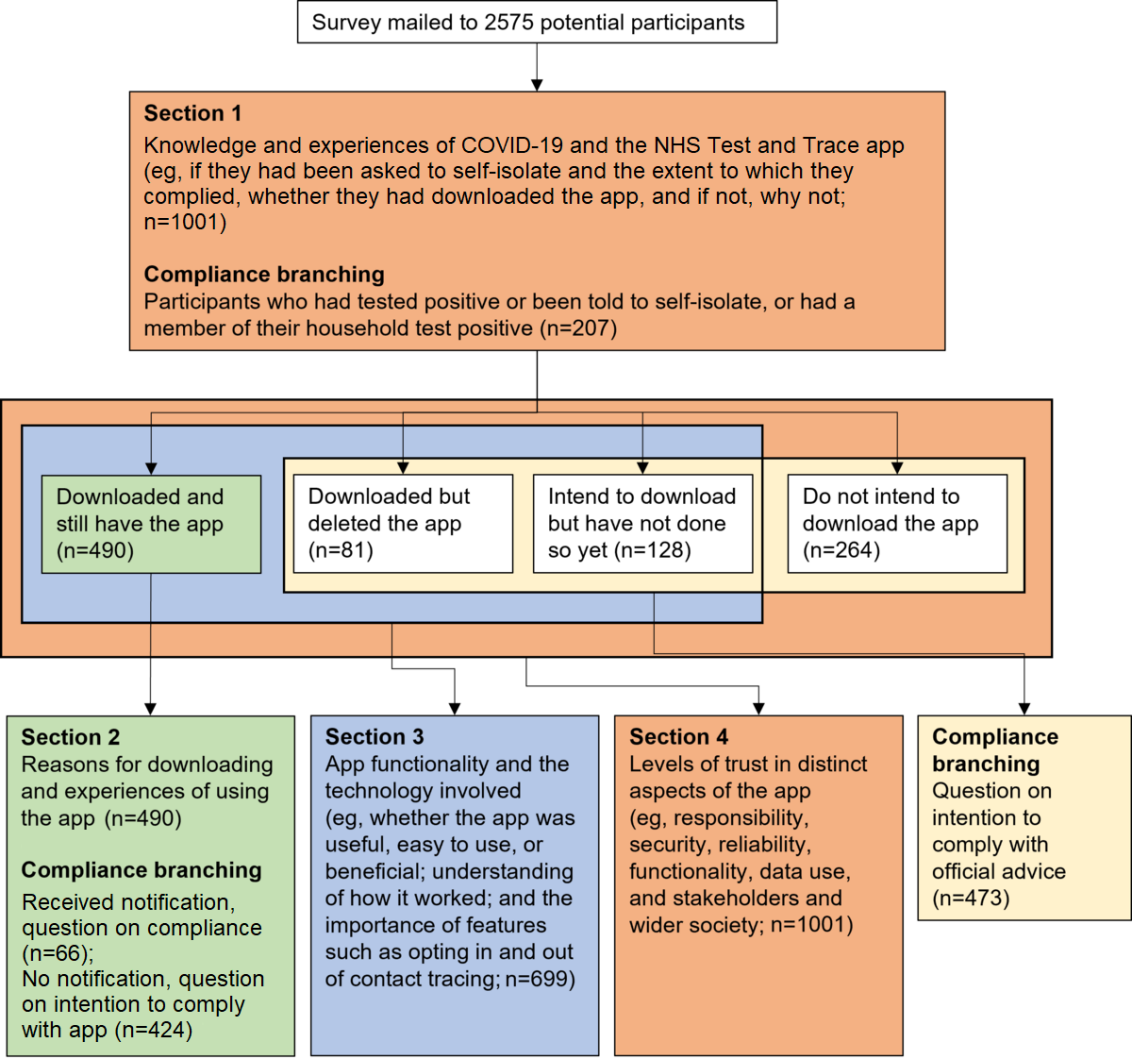
Illustration of major sections of the survey and subgroups identified for branching. NHS: National Health Service.

Figure 2 shows the survey flow and resulting major subpopulations that were used for branching. Section 1 of the survey asked participants to indicate what knowledge and experiences they had of COVID-19 and the NHS Test and Trace app; for example, if they had been asked to self-isolate and the extent to which they complied, whether they had downloaded the app, and if not, why not. Section 2 focused on those with the app and collected reasons for downloading and experiences of using the app. Section 3 asked about app functionality and the technology involved, including whether the app was useful, easy to use, or beneficial; understanding of how it worked; and the importance of features such as opting in and out of contact tracing. Section 4 asked about levels of trust in distinct aspects of the app, including responsibility, security, reliability, functionality, data use, and stakeholders and wider society.

### Statistical Analysis

Responses were analyzed using SPSS software (version 26; IBM Corp) and Excel (Microsoft 365). Summary statistics (ie, mean, median, SD, and IQR) or frequencies were extracted for all questions. Confidence intervals for proportions are given at the 95% level. Most questions were significantly nonnormal as shown by skewness and kurtosis, so nonparametric tests are appropriate. In the text, we report the sample mean, given with statistical test results, and the median response on a Likert-like scale. All inferential statistical analysis was carried out with *P*<.05 as the threshold for statistical significance. Missing data were reported as “no response” and were included in frequency calculations; missing data for inferential statistical analysis and the calculation of means were excluded.

Subgroup analyses using independent-samples Mann-Whitney *U* or chi-square tests were used to compare those who had been told to self-isolate with those who had not, White participants with BAME participants, and participants under 65 years with participants 65 years and over. Independent-samples Kruskal-Wallis tests were used to compare those who had the app, did not have the app, had deleted the app, or intended to download the app but had not yet done so. Post hoc tests were carried out to identify which groups had significant differences between them, with Bonferroni corrections to account for multiple testing. Due to use of the nonparametric versions of inferential statistics, and because the weighted values for the data all rounded to 1, weighting was not used. However, exploratory analysis revealed no difference in significance when using the parametric versions of tests.

## Results

### Adoption of the Test and Trace App

A total of 1001 participants took part in the study. Most participants (n=963, 96.2%; 95% CI 95.0%-97.4%) had heard of the NHS COVID-19 mobile phone app, of which 50.9% (490/963; 95% CI 47.8%-54.0%) had downloaded the app and still had it on their phone. A further 13.3% (128/963; 95% CI 11.2%-15.4%) had not yet downloaded it but intended to, 27.4% (264/963; 95% CI 24.6%-30.2%) did not intend to download it, and 8.4% (81/963; 95% CI 6.7%-10.1%) had downloaded it but since deleted it.

Among the 27.4% (264/963) of participants who did not intend to download the app, the most common reasons were a desire not to be tracked, not thinking it would be effective, not wanting to take part in contact tracing in that way, and lack of trust in those who built the app (Table 2). Of the 8.4% (81/963) who had decided to delete the app, this was mostly because they did not think it was effective or did not want to be tracked. Reasons given by the 13.3% (128/963) of participants who intended to download the app were mostly to help the NHS or to help protect their friends and family or themselves, as well as to reduce the spread of the virus and to help protect broader society (Table 3).

Of the 50.9% (490/963) of participants who had downloaded the app, 92.0% (451/490; 95% CI 90.3%-93.7%) had opened the app and had a look around, 66.7% (327/490; 95% CI 63.8%-69.6%) had used it for a venue check-in, 58.4% (286/490; 95% CI 55.3%-61.5%) had made use of the “check symptoms” section, 71.2% (349/490; 95% CI 68.4%-74.0%) had contact tracing always switched on, 20.4% (100/490; 95% CI 17.9%-22.9%) sometimes had contact tracing switched on, 1.8% (9/490; 95% CI 1.0%-2.6%) never turned contact tracing on, and 6.5% (32/490; 95% CI 5.0%-8.0%) did not know if contact tracing was enabled or not. The strongest reasons given for downloading the app were helping the NHS and protecting friends and family (Table 3).

**Table 2:** Reasons for not having the NHS Test and Trace app for those who do not intend to download it and those who downloaded but deleted the app.

Reasons for not having the app^a^	Participants who do not intend to download the app (n=264)			Participants who downloaded but deleted the app (n=81)	
	n (%)	95% CI	n (%)		95% CI
I don’t want to be tracked	105 (39.8)	36.8-42.8	22 (27.2)		24.4-30.0
I don’t think it will be effective	80 (30.3)	27.5-33.1	28 (34.6)		31.7-37.5
I choose/chose not to take part in contact tracing in this way	78 (29.5)	26.7-32.3	12 (14.8)		12.6-17.0
I don’t trust the people who built the app	72 (27.3)	24.5-30.1	14 (17.3)		15.0-19.6
The app doesn’t/didn’t work on my mobile phone	28 (10.6)	8.7-12.5	14 (17.3)		15.0-19.6
I don’t have a smartphone	27 (10.2)	8.3-12.1	N/A^b^		N/A
I don’t want to be told to self-isolate	16 (6.1)	4.9-7.6	9 (11.1)		9.2-13.0
None of the above	25 (9.5)	7.7-11.3	7 (8.6)		6.9-10.3
I wouldn’t/didn’t know how to use it	12 (4.5)	3.2-5.8	12 (14.8)		12.6-17.0
Don’t know	1 (0.1)	0-0.3	0 (0)		—^c^

^a^Multiple answers were allowed.

^b^N/A: not applicable, because participants do not have a smartphone, which is needed to download the app.

^c^The 95% CI value could not be calculated since there were no participants.

**Table 3:** Reasons for intention to download and for downloading the NHS COVID-19 app.

Reasons for intention to download and for downloading the app	Participants who intended to download the app^a^ (n=128)			The extent to which each reason was a motivation for downloading the app^b^ (n=490)	
	n (%)	95% CI	Mean (SD)		Median (IQR)
To help the National Health Service	84 (65.6)	62.7-68.5	4.42 (0.753)		5 (1)
To help protect my friends and family	80 (62.5)	59.5-65.5	4.36 (0.792)		5 (1)
To help protect myself	70 (54.7)	51.6-57.8	4.27 (0.883)		4 (1)
Because it will reduce the spread of the virus	55 (43.0)	39.9-46.1	4.11 (0.966)		4 (1)
To help protect broader society	46 (35.9)	32.9-46.1	4.2 (0.897)		4 (1)
Because I need it to check into venues	24 (18.8)	16.4-21.2	3.54 (1.179)		4 (1)
Because the government told me to	15 (11.7)	9.7-13.7	3.46 (1.177)		4 (1)
Because everyone else is	12 (9.4)	7.6-11.2	3.14 (1.177)		3 (2)
Because it is a requirement for my job	7 (5.5)	4.1-6.9	2.53 (1.361)		2 (3)
None of the above	2 (1.6)	0.8-2.4	N/A^c^		N/A

^a^Participants who intended to download the app were asked to select all reasons that applied.

^b^Participants who had the app were asked to what extent each reason was a motivation for downloading the app; responses were rated on a 5-point Likert scale from 1 (“strongly disagree”) to 5 (“strongly agree”).

^c^N/A: not applicable; this entry was not rated.

### Compliance With Official Advice and Self-isolation

**Figure 3:**
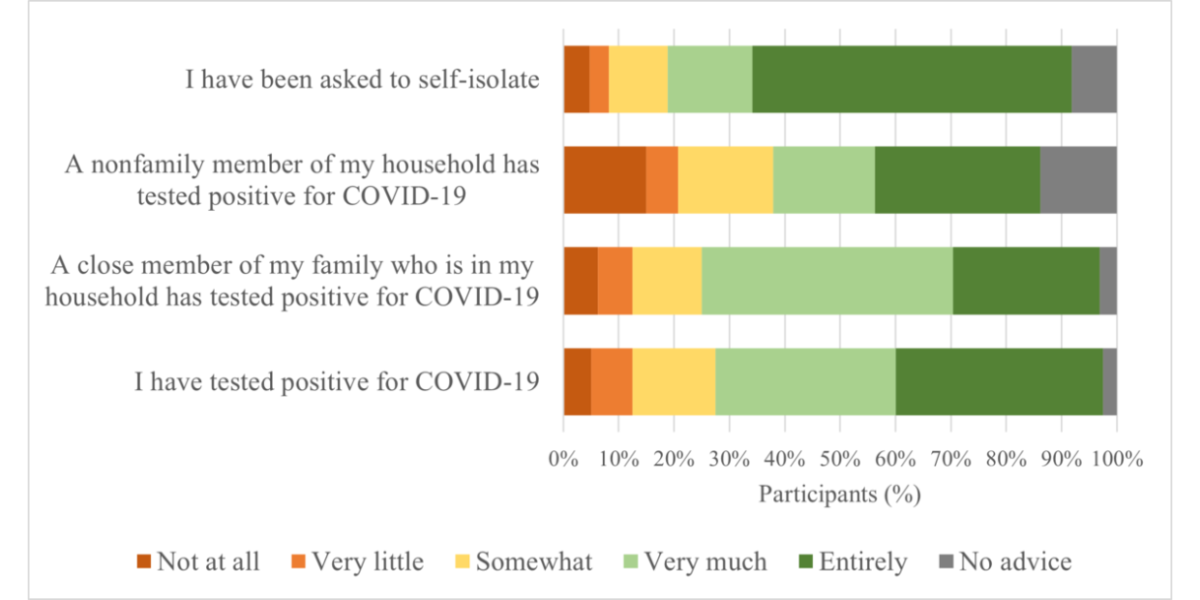
Compliance with official advice, dependent on the circumstances. Participants were asked "To what extent, if at all, did you follow any advice given to you?".

Out of 1001 participants, nearly half (n=434, 43.4%; 95% CI 40.3%-46.5%) had at least one of the following experiences: 4.0% (n=40; 95% CI 2.8%-5.2%) had tested positive for COVID-19, 14.1% (n=141; 95% CI 11.9%-16.3%) had a member of their household test positive, 27.9% (n=279; 95% CI 25.1%-30.7%) had another person close to them test positive, and 8.5% (n=85; 95% CI 6.8%-10.2%) had been asked to self-isolate in any form, whether via app or other means. Participants who had been affected or asked to self-isolate, or who had a member of their household who had (207/1001, 20.7%), were asked to indicate how much they had complied with any official advice they received, regardless of whether it was through the app or another source; the average response across all four experiences was “very much” (mean 3.88, SD 1.292). However, 10.6% (22/207; 95% CI 7.9%-11.5%) stated they did not receive any official advice at all, most often when a nonfamily member of their household had tested positive (12/87, 14%; 95% CI 11.7%-15.9%) (Figure 3). Note that this implies that some participants, therefore, did not consider being asked to self-isolate as “official advice.”

**Figure 4:**
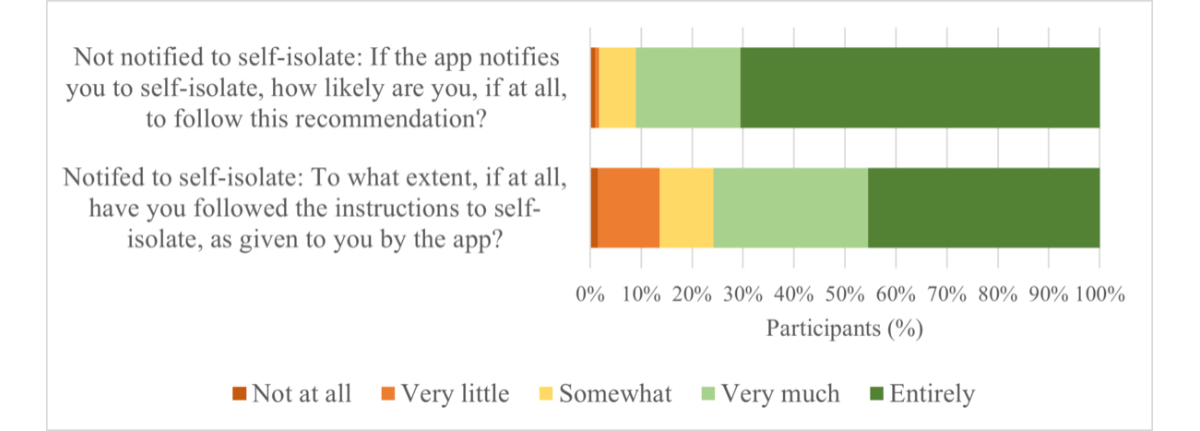
Compliance and intention to comply with app notifications to self-isolate. An independent-samples Mann-Whitney *U* test comparing those who had received advice to self-isolate (n=66) from the app to those who had not (n=418) shows that reported intention to comply with advice is significantly stronger than reported actual compliance (mean intention 4.59, SD 0.738; mean actual 4.06, SD 1.094; *U*=17673.0; *P*<.001).

Of the 47.3% (473/1001) of participants who did not currently have the app, most stated they would either very much or entirely follow advice to self-isolate if they received a call (352/473, 74.4%; 95% CI 71.7%-77.1%). Of participants with the app (490/963, 50.9%), 13.5% (66/490; 95% CI 11.4%-15.6%) had been notified to self-isolate, and 45% (30/66; 95% CI 42.4%-48.6%) of those said that they had entirely followed the recommendation. However, of the 85.3% (418/490; 95% CI 83.1%-87.5%) who stated that they had not been notified by the app to self-isolate, 70.6% (295/418; 95% CI 73.4%-78.6%) said they would entirely follow a recommendation from the app if they received one (Figure 4). An independent-samples Mann-Whitney *U* test comparing those who had received advice to self-isolate from the app (66/490, 13.5%) to those who had not (418/490, 85.3%) shows that reported intention to comply with advice is significantly stronger than reported actual compliance (mean_intention_ 4.59, SD 0.738; mean_actual_ 4.06, SD 1.094; *U*=17673.0; *P*<.001).

### Understanding and Attitudes Toward the App

Of participants who currently have the app, have deleted it, or intended to download it (699/963, 72.6%), most stated that they thought that decisions to send a notification to self-isolate were made by both humans and the app (379/699, 54.2%; 95% CI 51.1%-57.3%); only 19.5% (136/699; 95% CI 17.0%-22.0%) thought they were made by the app only. Participants with the app agreed that they knew how the app worked, that it was easy to use, that it was useful to them and to wider society, that the regulations surrounding the app were sufficient, that it was important that they could get explanations and verify information from the app, that they could speak to a person about any advice they receive, and that they have the option to opt out of contact tracing if they chose to. They were neutral about data concerns and about whether they had a choice in downloading the app. They tended to disagree that they had been frustrated by a notification from the app. Independent-samples Kruskal-Wallis tests showed that participants who had deleted the app (81/963, 8.4%) felt significantly less than those who still have the app (490/963, 50.9%) that they understood the app (χ^2^_2_=16.1, *P*=.003), that it was useful to them (χ^2^_2_=26.2, *P*<.001) or wider society (χ^2^_2_=29.7, *P*<.001), that regulations were sufficient (χ^2^_2_=12.9, *P*=.003), or that it was easy to use (χ^2^_1_=41.2, *P*<.001). It was also significantly less important to them that they could verify app notifications (χ^2^_2_=18.3, *P*<.001). However, they showed significantly more concern about how their data were used (χ^2^_2_=25.7, *P*<.001) and were more likely to have been frustrated by a notification from the app (χ^2^_1_=18.3, *P*<.001). None of the other statements (Table 4) were significantly different.

**Table 4:** Levels of agreement with statements related to the technology and ecosystem surrounding the NHS COVID-19 app.

Statements^a^	Participants who still have the app (n=490)			Participants who deleted the app (n=81)			*P* value^b^
	Mean (SD)	Median (IQR)	Mean (SD)		Median (IQR)		
I understand how the NHS COVID-19 app works	3.99 (0.850)	4 (1)	3.57 (1.036)		4 (1)	.003	
I am concerned about how my data will be used by the app	3.04 (1.250)	3 (2)	3.73 (1.037)		4 (1)	<.001	
The app is useful to me personally	3.84 (0.944)	4 (2)	3.19 (1.174)		3 (2)	<.001	
The app is useful to wider society	4.11 (0.874)	4 (1)	3.47 (1.096)		4 (1)	<.001	
It is important to me that I can get an explanation for any information given to me by the app	4.01 (0.801)	4 (1)	3.72 (1.028)		4 (2)	.052	
It is important to me that I can verify that notifications from the app are authentic	4.14 (0.823)	4 (1)	3.60 (1.137)		4 (1)	<.001	
The regulations governing the creation of the app are sufficient	3.72 (0.934)	4 (1)	3.37 (1.089)		3 (1)	.003	
It is important to me to be able to speak to a person about any advice given by the app	3.71 (0.982)	4 (1)	3.64 (1.099)		4 (1)	>.99	
It is important to me that I can opt in and opt out of contact tracing	3.50 (1.166)	4 (1)	3.58 (0.947)		4 (1)	>.99	
The app is easy to use	4.18 (0.815)	4 (1)	3.42 (1.082)		4 (1)	<.001	
I felt that I had no choice but to download the app	2.98 (1.273)	3 (2)	3.27 (1.162)		3 (2)	.06	
I have felt frustrated as a result of a notification from the app	2.59 (1.279)	2 (2)	3.28 (1.154)		3 (2)	<.001	

^a^Statements were rated on a 5-point Likert scale from 1 (“strongly disagree”) to 5 (“strongly agree”).

^b^Independent-samples Kruskal-Wallis tests were carried out with a significance level of *P*<.05 and post hoc tests to indicate which groups had significant differences between them, with Bonferroni correction to account for multiple tests. Additional differences, including participants who downloaded and still have the app and those who intend to download the app, are in Table S2 in Multimedia Appendix 1.

### Trust in Test and Trace

While those who still have the app (490/963, 50.9%) tended to agree that they had trust in various aspects of the app (Table 5), independent-samples Kruskal-Wallis tests showed that those who chose not to download the app (264/963, 27.4%) had significantly less trust, feeling neutral regarding trusting that the data were used responsibly (χ^2^_3_=222.2, *P*<.001) and stored securely (χ^2^_3_=236.3, *P*<.001), that the app does what it is supposed to do (χ^2^_3_=273.9, *P*<.001), and that the app is basically trustworthy (χ^2^_3_=243.1, *P*<.001). They were also significantly less trusting of others, feeling neutral about whether they trusted others to download the app (χ^2^_3_= 128.8, *P*<.001) or to self-isolate if they were told to (χ^2^_3_=74.1, *P*<.001). Participants who chose not to download the app were also significantly more likely not to trust that their data would be deleted when the app said it would be (χ^2^_3_=251.1, *P*<.001) or that the app was reliable (χ^2^_3_=277.7, *P*<.001). Trust was thought to be important for all participants to feel comfortable using the app, although significantly less so for those who chose not to download it (χ^2^_3_=24.0, *P*<.001).

**Table 5:** Levels of agreement with statements related to trust in the NHS COVID-19 app.

Statements^a^	Participants who have the app (n=490)		Participants who do not intend to download the app (n=264)		*P* value^b^
	Mean (SD)	Median (IQR)	Mean (SD)	Median (IQR)	
I trust that the data collected by the app are used responsibly	3.98 (0.888)	4 (1)	2.63 (1.224)	3 (2)	<.001
I trust that the data collected by the app are stored securely	3.93 (0.916)	4 (2)	2.56 (1.149)	3 (1)	<.001
I feel that the app is reliable	3.89 (0.919)	4 (2)	2.42 (1.062)	2 (1)	<.001
I trust that the app will do what it is supposed to do	3.97 (0.880)	4 (1)	2.53 (1.136)	3 (1)	<.001
I think the NHS COVID-19 app is basically trustworthy	4.03 (0.875)	4 (1)	2.73 (1.129)	3 (1)	<.001
I think that most other people will download the app	3.58 (1.026)	4 (1)	2.65 (1.086)	3 (1)	<.001
I trust that most other people will self-isolate if told to do so by the app	3.54 (1.113)	4 (1)	2.80 (1.106)	3 (2)	<.001
I trust that my data will be deleted when the app says they will be	3.93 (0.892)	4 (2)	2.48 (1.196)	2 (2)	<.001
It is important to me that I trust the app in order to use it	4.15 (0.736)	4 (1)	3.86 (1.145)	4 (2)	<.001

^a^Statements were rated on a scale from 1 (“strongly disagree”) to 5 (“strongly agree”).

^b^Independent-samples Kruskal-Wallis tests were carried out with a significance level of *P*<.05 and post hoc tests to indicate which groups had significant differences between them, with Bonferroni correction to account for multiple tests. Additional differences, including participants who deleted or intended to download the app, are in Table S3 in Multimedia Appendix 1.

While those who still had the app (490/963, 50.9%) agreed that they trusted most of the stakeholders involved in the NHS Test and Trace system (Table 6), independent-samples Kruskal-Wallis tests showed that those who chose not to download the app (264/963, 27.4%) were significantly more neutral about big tech companies (χ^2^_3_=82.1, *P*<.001), small hospitality venues (χ^2^_3_=34.8, *P*<.001), large hospitality venues (χ^2^_3_=56.1, *P*<.001), and their local council (χ^2^_3_=61.7, *P*<.001), and significantly more negative about their trust in the UK government (χ^2^_3_=61.7, *P*<.001). Those with the app were neutral about their trust in private contractors, while those without the app were significantly more negative (χ^2^_3_=85.6, *P*<.001). Finally, both groups tended to agree that they trusted the NHS, but those without the app significantly less so (χ^2^_3_=78.9, *P*<.001).

**Table 6:** Levels of trust in stakeholders involved in the NHS Test and Trace system among participants.

Statements^a^	Participants who have the app (n=490)			Participants who do not intend to download the app (n=264)			*P* value^b^
	Mean (SD)	Median (IQR)	Mean (SD)		Median (IQR)		
I trust the big tech companies, such as Google and Apple	3.42 (0.996)	4 (1)	2.65 (1.134)		3 (1)	<.001	
I trust private contractors, such as Serco	3.07 (1.086)	3 (2)	2.29 (1.021)		2 (2)	<.001	
I trust small hospitality venues, such as independent pubs and cafés	3.62 (0.864)	4 (1)	3.13 (1.125)		3 (2)	<.001	
I trust larger hospitality venues, such as chain restaurants	3.52 (0.923)	4 (1)	2.91 (1.068)		3 (2)	<.001	
I trust the UK Government	3.32 (1.213)	4 (2)	2.39 (1.181)		2 (2)	<.001	
I trust my local council	3.51 (0.968)	4 (1)	2.86 (1.096)		3 (2)	<.001	
I trust the National Health Service	4.33 (0.774)	4 (1)	3.72 (1.102)		4 (2)	<.001	

^a^Statements were rated on a 5-point Likert scale from 1 (“strongly disagree”) to 5 (“strongly agree”).

^b^Independent-samples Kruskal-Wallis tests were carried out with a significance level of *P*<.05 and post hoc tests to indicate which groups had significant differences between them, with Bonferroni correction to account for multiple tests. Additional differences, including participants who deleted or intended to download the app, are in Table S4 in Multimedia Appendix 1.

### Vulnerable Groups

Of the 11.5% (115/1001) of participants who identified as BAME, 53.0% (61/115; 95% CI 49.9%-56.1%) had any close experience of COVID-19, including friends and family receiving diagnoses, compared to 42.3% (371/876; 95% CI 39.2%-45.4%) of White participants (Table 7). Chi-square tests showed that significantly more BAME participants had a member of their household test positive, (χ^2^_1_=10.0, *P*<.05). While more BAME participants had tested positive or had another person close to them test positive than White participants, the differences were not significant; a similar proportion had been asked to self-isolate. Significantly fewer BAME participants than White participants had downloaded the app (χ^2^_1_=4.7, *P*<.05) and more had deleted the app (χ^2^_1_=4.5, *P*<.05); while fewer BAME participants did not intend to download the app and more intended to download it, neither difference was significant (Table 8). BAME participants agreed significantly less that they downloaded the app to help the NHS (mean_BAME_ 4.02, SD 1.000; mean_White_4.46, SD 0.710; *U*=7803.5; *P*=.001) and significantly more that it was a requirement for their job (mean_BAME_ 3.19, SD 1.437; mean_White_ 2.45, SD 1.341; *U*=13734.0; *P*<.001); no other reasons showed a difference.

**Table 7:** Experiences of COVID-19 among vulnerable populations (BAME participants and those 65 years and over) compared to other populations (White participants and those under 65 years).

Experiences of COVID-19	White participants (n=876)			BAME^a^ participants (n=115)			Participants under 65 years (n=874)			Participants 65 years and over (n=127)	
	n (%)	95% CI	n (%)		95% CI	n (%)		95% CI	n (%)		95% CI
Any close experience of COVID-19	371 (42.3)	39.2-45.4	61 (53.0)		49.9-56.1	407 (46.6)		43.5-49.7	28 (22.0)		19.4-24.6
Tested positive	32 (3.7)	2.5-4.9	8 (7.0)		5.4-8.6	39 (4.5)		3.2-5.8	1 (0.9)		0.3-1.5
Member of household tested positive	112 (12.7)	10.6-14.8	29 (25.2)		22.5-27.9	136 (15.6)		13.4-17.8	5 (3.9)		2.7-5.1
Another close person tested positive	241 (27.5)	24.7-30.3	36 (31.3)		28.4-34.2	265 (30.3)		27.5-33.1	14 (11.0)		9.1-12.9
Asked to self-isolate	76 (8.7)	7.0-10.4	7 (7.0)		5.4-8.6	74 (8.5)		6.8-10.2	11 (8.8)		7.0-10.4

^a^BAME: Black, Asian, and minority ethnic.

**Table 8:** Downloads of the NHS COVID-19 app among vulnerable populations (BAME participants and those 65 years and over) compared to other populations (White participants and those under 65 years).

Download status	White participants (n=876)		BAME^a^ participants (n=115)			Participants under 65 years (n=874)			Participants 65 years and over (n=127)	
	n (%)	95% CI	n (%)	95% CI	n (%)		95% CI	n (%)		95% CI
Downloaded	440 (50.2)	47.1-53.3	48 (41.7)	38.6-44.8	424 (48.5)		45.4-51.6	66 (52.0)		48.9-55.1
Downloaded then deleted	65 (7.4)	5.8-9.0	16 (13.9)	11.8-16.0	79 (9.0)		7.2-10.8	2 (1.6)		0.8-2.4
Not downloaded but intend to	105 (12.0)	10.0-14.0	19 (16.5)	14.2-18.8	115 (13.2)		11.1-15.3	13 (10.2)		8.3-12.1
Do not intend to download	236 (26.9)	24.2-29.6	24 (20.9)	18.4-23.4	220 (25.2)		22.5-27.9	44 (34.6)		32.6-38.6

^a^BAME: Black, Asian, and minority ethnic.

Of the 12.7% (127/1001) of participants who were 65 years of age or over, only 22.0% (28/127; 95% CI 19.4%-24.6%) had any close experience of COVID-19 compared to 46.6% (407/874; 95% CI 43.5%-49.7%) of participants under 65 years. Chi-square tests showed that significantly more participants under 65 years than those 65 years and over had a member of their household test positive (χ^2^_1_=10.4, *P*<.05) or another person close to them (χ^2^_1_=4.7, *P*<.05), with no significant difference in testing positive for COVID-19 or being asked to self-isolate. Significantly more participants 65 years and over had downloaded the app (χ^2^_1_=7.0, *P*<.05), fewer participants 65 years and over had deleted it (χ^2^_1_=8.2, *P*<.05), but significantly more participants 65 years and over, compared to those under 65 years, did not intend to download the app (χ^2^_1_=13.5, *P*<.05); similar proportions intended to download the app. Participants 65 years and over agreed significantly more that they downloaded the app to help the NHS (mean_≥65_ 4.59, SD 0.656; mean_<65_ 4.39, SD 0.764; *U*=16164.0; *P*=.02) and to help protect their friends and family (mean_≥65_ 4.50, SD 0.846; mean_<65_ 4.34, SD 0.782; *U*=16220.0; *P*=.02) or broader society (mean_≥65_ 4.45, SD 0.786; mean_<65_ 4.16, SD 0.910; *U*=16630.5; *P*=.008); however, they were less likely to agree that it was needed for them to check into venues (mean_≥65_ 3.15, SD 1.256; mean_<65_ 3.60, SD 1.157; *U*=11089.5; *P*=.005) or for their job (mean_≥65_ 1.94, SD 1.188; mean_<65_ 2.63, SD 1.364; *U*=9874.0; *P*<.001); no other reasons showed a difference.

Of participants who did not have the app, intention to comply with a phone call asking them to self-isolate was the same between both BAME and White participants (mean_BAME_ 3.93, SD 1.201; mean_White_ 4.14, SD 1.192; *U*=13909.0; *P*=.06) and between participants 65 years and over and those under 65 years (mean_≥65_ 4.39, SD 1.000; mean_<65_ 4.08, SD 1.207; *U*=10573.0; *P*=.11). Of those with the app who had been notified to self-isolate, there was no significant difference in compliance between populations (mean_BAME_ 3.67, SD 1.234; mean_White_ 4.18, SD 1.034; *U*=291.5; *P*=.14; mean_≥65_ 4.25, SD 1.500; mean_<65_ 4.05, SD 1.078; *U*=150.0; *P*=.51), but of those who had not been notified, White participants reported a significantly higher intention to comply (mean_BAME_ 4.26, SD 0.855; mean_White_ 4.63, SD 0.700; *U*=4544.5; *P*=.006), as did participants 65 years and over (mean_≥65_ 4.90, SD 0.349; mean_<65_ 4.54, SD 0.774; *U*=13838.5; *P*<.001).

BAME participants had a significantly lower understanding of how decisions were made by the app (χ^2^_2_=9.2, *P*<.05) (Table 9); more participants thought it was either humans only or humans and the app, while far fewer understood that it was only the app. While more participants 65 years and over than those under 65 years also felt that decisions were made entirely by humans, fewer felt decisions were made by both humans and the app, and more of them correctly identified that decisions were made entirely by the app; there was no significant difference between the age groups (Table 9).

**Table 9:** Beliefs about how decisions are made by the NHS COVID-19 app among vulnerable populations (BAME participants and those 65 years and over) compared to other populations (White participants and those under 65 years).

Beliefs about how decisions are made by the app	White participants (n=876)		BAME^a^ participants (n=115)			Participants under 65 years (n=874)			Participants 65 years and over (n=127)	
	n (%)	95% CI	n (%)	95% CI	n (%)		95% CI	n (%)		95% CI
Humans only	156 (17.8)	15.4-20.2	26 (22.6)	20.0-25.2	159 (18.2)		15.8-20.6	25 (19.7)		17.2-22.2
Both humans and the app	324 (37.0)	34.0-40.0	51 (44.3)	41.2-47.4	343 (39.2)		36.2-42.2	36 (28.3)		25.5-31.1
App only	130 (14.8)	12.6-17.0	6 (5.2)	3.8-6.6	116 (13.3)		11.2-15.4	20 (15.7)		13.4-18.0

^a^BAME: Black, Asian, and minority ethnic.

BAME participants were more concerned about how their data would be used (mean_White_ 3.07, SD 1.232; mean_BAME_ 3.58, SD 1.091; *U*=31052.5; *P*=.001), felt more strongly that they had no choice but to download the app (mean_White_ 2.95, SD 1.267; mean_BAME_ 3.59, SD 1.065; *U*=20857.0; *P*<.001), and felt frustrated as a result of a notification from the app (mean_White_ 2.59, SD 1.263; mean_BAME_ 3.42, SD 1.219; *U*=21961.5; *P*<.001). They felt less strongly that the app was easy to use (mean_White_ 4.11, SD 0.871; mean_BAME_ 3.75, SD 1.039; *U*=12844.5; *P*=.004) or that it was useful to wider society (mean_White_ 4.05, SD 0.885; mean_BAME_ 3.75, SD 0.960; *U*=21371.5; *P*=.01). Participants 65 years and over were less concerned about how their data would be used (mean_≥65_ 2.72, SD 1.269; mean_<65_ 3.20, SD 1.216; *U*=20663.0; *P*=.009), less likely to feel that they had no choice but to download the app (mean_≥65_ 2.68, SD 1.215; mean_<65_ 3.07, SD 1.260; *U*=14010.0; *P*=.01), and less likely to feel frustrated as a result of a notification from the app (mean_≥65_ 2.09, SD 1.129; mean_<65_ 2.77, SD 1.283; *U*=11854.5; *P*<.001). It was also less important to them that they could opt in and opt out of contact tracing (mean_≥65_ 3.18, SD 1.119; mean_<65_ 3.56, SD 1.101; *U*=19243.0; *P*<.001). There were no other significant differences in attitudes.

BAME participants felt it was less important that they trusted the app (mean_White_ 4.04, SD 0.898; mean_BAME_ 3.79, SD 0.996; *U*=43247.5; *P*=.008). They had more trust in the big tech companies (mean_White_ 3.14, SD 1.106; mean_BAME_ 3.43, SD 1.109; *U*=57731.5; *P*=.008) and private contractors (mean_White_ 2.78, SD 1.099; mean_BAME_ 3.24, SD 1.081; *U*=61597.5; *P*<.001), but less trust in the NHS (mean_White_ 4.12, SD 0.954; mean_BAME_ 3.97, SD 0.912; *U*=44705.5; *P*=.04). Conversely, participants 65 years and over had less trust in the big tech companies (mean_≥65_ 2.97, SD 1.133; mean_<65_ 3.21, SD 1.100; *U*=49432.0; *P*=.04) and private contractors (mean_≥65_ 2.53, SD 1.133; mean_<65_ 2.88, SD 1.092; *U*=46020.0; *P*=.001), but more trust in the UK government (mean_≥65_ 3.24, SD 1.269; mean_<65_ 2.97, SD 0.945; *U*=62275.5; *P*=.02). There were no other significant differences in trust.

## Discussion

### Principal Findings and Comparison With Prior Work

Just over half of those surveyed had downloaded the app, agreeing with other estimates for the United Kingdom [29]. Reasons for app uptake were predominantly to help the NHS, protect others, and reduce the spread of the virus, broadly agreeing with previous research [2,4]. Older adults had more community-minded and altruistic attitudes, being more likely to download the app to help the NHS, friends, family, and society, but they also had less intention to download the app. However, almost 1 in 9 of those who initially downloaded the app eventually deleted it, especially BAME participants, although the number of these participants who had downloaded and kept the app was similar to that of White participants. However, this increased deletion is a particular concern, as their vulnerability is reflected in the finding that twice as many BAME participants had tested positive or had a household member test positive for COVID-19.

In line with previous studies [4,10,13-15], reasons for not downloading or deleting the app were related to not wanting to be tracked, a feeling that it would be ineffective, and a lack of trust in the people who built the app. People who decided to delete the app were more likely than those who kept it to feel that it was not useful or easy to use, as expected from the TAM2 [32]; they also felt they understood less about how it worked, were more concerned about how their data would be used, and were more likely to have been frustrated by a notification from the app. This shows how important a trustworthy user experience is for the adoption of contact tracing apps. BAME participants who had the app also had higher levels of concern about their data, felt the app was less easy to use and was less useful to society, and were more likely to have been frustrated by the app; this could lead to them deleting the app in the future. Those involved in the design of contact tracing should pay particular attention to the needs of BAME app users. On the other hand, older adults had less concern about their data and less frustration. Engaging users in the development, implementation, and evaluation of contact tracing can help to maximize engagement and technology acceptance, according to the RRI framework [7,8], helping designers to consider wider social implications of a technology and how real-world use might differ from usage within a trial or with a prototype. Working together with users to anticipate concerns and develop solutions can be an effective mechanism to achieve the adoption of digital solutions.

The feeling that there was a lack of choice in using the app was stronger among BAME participants and lowest among older adults; BAME participants were also more likely to state that they had to download the app for their job. Participants required a level of control over the app, feeling that it was important to get explanations, verify and speak to people about notifications, and be able to opt out of contact tracing; interestingly, the latter was less important for older adults. Most people stated that they would be or had been highly compliant with advice to self-isolate, although intention was significantly higher than actual reported compliance, especially among older adults. Actual reported compliance was similar across the different groups, although our findings suggest that White people tended to overstate their intention to comply. This finding may be impacted by the reduced trust in the government, as previously discussed, as well as a perceived lack of incentives offered for compliance [33]. It is also interesting that when asked how much they complied with “official advice,” some participants who had been asked to self-isolate, by the app or otherwise, occasionally answered that they had not received any official advice. This implies that the instruction to self-isolate was perceived as being a suggestion rather than holding any authority, which may, in turn, have led to participants not taking the same precautions as they would have if they felt that the instruction to self-isolate was an official request.

Trust in the app was moderate. Participants who did not download the app had significantly lower trust in the app, especially in whether their data would be deleted and whether the app was reliable. They also had significantly lower trust in other users and in stakeholders surrounding the app, suggesting that trust is an important consideration in the design of contact tracing apps. Trust in the government was particularly low and may be a factor in adoption of app-based contact tracing [2,4,34,35], although it was higher among older adults, who were more concerned about big tech and private contractors. The BAME population had more trust in the big tech companies and private contractors but less trust in the NHS. BAME participants were also significantly less likely to download the app to help the NHS. A recent study on health-related quality of life revealed inequalities within English ethnic minorities [36], including poor primary care experiences, inadequate support from local services, and low patient self-confidence. This indicates that government slogans like “protect the NHS” may not have the intended effect on BAME communities and should be rethought to be more inclusive. However, reasons for a lack of trust on the part of BAME people in the governments and institutions in the United Kingdom are likely linked to persistent issues of structural racism [37] and, thus, are unlikely to be changed through singular measures alone, such as government messaging.

Finally, although participants felt they understood how the app worked, the results show that most people do not know that decisions about notifications are entirely made by the app [27,29], without human involvement. Understanding was particularly low in BAME communities. This lack of understanding may affect uptake and continued use, as it may negatively impact trust and, consequently, the app’s popularity, perceived validity, and reliability [38]. At the same time, perceived human intervention may falsely increase trust in the app, as a completely automated system is likely to be recognized as having an unfair impact on the population, limiting freedom without taking into consideration personal circumstances [39].

### Limitations and Future Work

While the sample was representative of the UK population in terms of age, gender, region, and ethnicity, some demographics that were not measured, such as income and political leaning, may have affected the results. Additionally, this representativeness means that the sample sizes for the vulnerable populations, although proportionate to population, were small compared to the overall sample. Future work should consider enriching the sample with greater numbers of participants from minority populations in order to capture their views more thoroughly. Additionally, while the sample was drawn from an online panel, this bias toward the online population was considered acceptable for this study, as the focus was on use of a smartphone app, which implied internet access. Recent estimates suggest that 92% of adults in the United Kingdom are recent internet users, including 54% of those over 75 years of age and 81% of disabled adults; only 6.3% of adults had never used the internet [40]. However, this does mean that potential respondents who do not have access to, or the ability to use, the internet—as were individuals who were not part of the online panel—were unable to take part in this study. While out of scope for this paper, this should be examined in future studies, as such issues could disproportionately affect vulnerable communities.

Future work should also consider multivariable analyses to account for the demographics of participants, to aid in explaining the differences found between vulnerable subgroups. For example, it is possible that lower trust in the government or the NHS might be driven by factors such as age, gender, or education. Similarly, further investigation of other groups could be beneficial, for example, to test the effects of having tested positive on their opinions or behaviors. There was a slightly higher proportion of participants who had tested positive than the national proportion (4% vs 3%), and quite a few participants reported they had been otherwise affected by the virus; it is possible that some participants were drawn to the study for this reason. However, this group was not excluded or highlighted in this paper due to the overall low numbers of self-reported positive cases among respondents.

Finally, as with all self-report studies, in addition to the potential oversubscription of closely affected participants, there is a possibility of other reporting biases in this study; for example, social desirability bias and overreporting of compliance to self-isolation advice. However, as described above, a nontrivial number of participants did report not complying at all or not intending to comply, although it does seem there is a tendency to overstate intentions. It would be interesting to relate this to actual recorded behavior with regard to self-isolation.

### Conclusions

This paper adds to the existing evidence surrounding digital contact tracing by reporting on an investigation of acceptance of a live app, which had been available to download for almost 3 months at the time of the study. Based on the TAM with the added factor of trust, an online survey was carried out looking at use of and attitudes toward the United Kingdom’s NHS Test and Trace app, NHS COVID-19, among a representative sample of participants, including subgroup analysis of participants 65 years and over and members of the BAME community as potentially vulnerable users. Results indicate that uptake was limited to around 1 in 2 persons. Stated reasons for adoption predominantly surrounded a desire to help the NHS, friends and family, and society, especially among older adults, although BAME respondents agreed significantly less that their reason was to help the NHS. However, of those with the app, only one-fifth understood that the decision to send self-isolation notifications was made by the app without human involvement; in addition, there were a range of significantly more negative views among BAME participants. Respondents without the app reported significantly lower trust and more negative views toward the app.  In cohort with other studies, the evidence shows that there are considerable barriers to the uptake of digital contact tracing apps, and these differ across different populations. It is important to consider especially potentially vulnerable groups to ensure that interventions such as these are effective. Potential users must be engaged in the design to enhance uptake and acceptance of test and trace apps, focusing particularly on groups that might be hard to reach or may have different attitudes toward acceptance. 

## Appendix

Multimedia Appendix 1 Demographics and additional statistics from the study.

## Abbreviations

BAME: Black, Asian, and minority ethnic
NHS: National Health Service
RRI: Responsible Research and Innovation
TAM: Technology Acceptance Model
TAM2: extended Technology Acceptance Model
