# Supplementary material for: Public Adoption of and Trust in the NHS COVID-19 Contact Tracing App in the United Kingdom: Quantitative Online Survey Study
Source: J Med Internet Res. 2021 Sep 17;23(9):e29085. doi: 10.2196/29085 (PMC8451731; doi:10.2196/29085)
Supplement: Multimedia Appendix 1 [file jmir_v23i9e29085_app1.docx]

**Table S1. Summary of main participant demographics**

|  | Response options | % | Freq. |
| --- | --- | --- | --- |
| **Age** | 16-24 | 15.2 | 152 |
|  | 25-34 | 18.9 | 189 |
|  | 35-44 | 18.0 | 180 |
|  | 45-54 | 19.0 | 190 |
|  | 55-64 | 16.3 | 163 |
|  | 65-75 | 12.7 | 127 |
| **Gender** | Male | 50.0 | 501 |
|  | Female | 50.0 | 500 |
| **Current Employment** | Employed full-time | 45.6 | 456 |
|  | Employed part-time | 16.0 | 160 |
|  | Self employed | 5.0 | 50 |
|  | Unemployed but looking for a job | 5.1 | 51 |
|  | Unemployed and not looking for a job/Long-term sick or disabled | 5.8 | 58 |
|  | Full-time parent, homemaker | 6.6 | 66 |
|  | Retired | 11.7 | 117 |
|  | Student/Pupil | 4.3 | 43 |
| **Employment status (autocoded)** | Working | 66.5 | 666 |
|  | Not working | 33.5 | 335 |
| **Highest Educational Qualification** | Primary school | 0.2 | 2 |
|  | Secondary school (age under 15 years old) | 6.4 | 64 |
|  | GNVQ / GSVQ / GCSE/ SCE standard | 24.1 | 241 |
|  | NVQ1, NVQ2 | 8.4 | 84 |
|  | NVQ3/ SCE Higher Grade/ Advanced GNVQ/ GCE A/AS or similar | 22.4 | 224 |
|  | NVQ4 / HNC / HND / Bachelor's degree or similar | 29.0 | 290 |
|  | NVQ5 or post-graduate diploma | 9.6 | 96 |
| **Education (autocoded)** | Up to GSCE | 30.7 | 307 |
|  | Post-GSCE | 69.4 | 694 |
| **Ethnic identity** | English / Welsh / Scottish / Northern Irish / British | 83.7 | 838 |
|  | Irish | 0.7 | 7 |
|  | Any other White background | 3.1 | 31 |
|  | White and Black Caribbean | 0.9 | 9 |
|  | White and Black African | 0.4 | 4 |
|  | White and Asian | 0.5 | 5 |
|  | Any other Mixed / multiple ethnic background | 0.3 | 3 |
|  | Indian | 2.1 | 21 |
|  | Pakistani | 1.7 | 17 |
|  | Bangladeshi | 0.4 | 4 |
|  | Chinese | 0.9 | 9 |
|  | Any other Asian background | 1.1 | 11 |
|  | African | 1.8 | 18 |
|  | Caribbean | 0.7 | 7 |
|  | Any other Black / African / Caribbean background | 0.2 | 2 |
|  | Arab | 0.2 | 2 |
|  | Any other ethnic group | 0.3 | 3 |
|  | Prefer not to answer/consent not granted | 1.0 | 10 |
| **Ethnicity (autocoded)** | White | 87.5 | 876 |
|  | BAME | 11.5 | 115 |
|  | Not stated | 1.0 | 10 |
| **Location** | North East | 4.6 | 46 |
|  | North West | 11.0 | 110 |
|  | Yorkshire and The Humber | 8.3 | 83 |
|  | West Midlands | 8.7 | 87 |
|  | East Midlands | 7.2 | 72 |
|  | East of England | 9.2 | 92 |
|  | South West | 8.0 | 80 |
|  | South East | 14.1 | 141 |
|  | Greater London | 13.6 | 136 |
|  | Wales | 4.8 | 48 |
|  | Scotland | 8.5 | 85 |
|  | Northern Ireland | 2.1 | 21 |

**Table S2. Levels of agreement with statements related to the technology and ecosystem surrounding the NHS Covid-19 app, n=699**

Independent samples Kruskall-Wallis test with post-hoc examination using the Bonferroni correction, significance level *P*<.05, shows significant differences indicated by:

^a^ between Downloaded and still have the app and Downloaded and deleted the app

^b^ between Downloaded and still have the app and Intend to download the app

^c^ Between Downloaded and deleted the app and Intend to download the app

|  | **Median** | **IQR** | **Mean** | **SD** |
| --- | --- | --- | --- | --- |
| **I understand how the NHS Covid-19 app works ^a^** |  |  |  |  |
| Overall sample statistic | 4 | 1 | 3.90 | 0.883 |
| Downloaded and still have the app | 4 | 1 | 3.99 | 0.850 |
| Downloaded and deleted the app | 4 | 1 | 3.57 | 1.036 |
| Intend to download the app | 4 | 1 | 3.79 | 0.848 |
| **I am concerned about how my data will be used by the app ^a b^** |  |  |  |  |
| Overall sample statistic | 3 | 2 | 3.18 | 1.228 |
| Downloaded and still have the app | 3 | 2 | 3.04 | 1.250 |
| Downloaded and deleted the app | 4 | 1 | 3.73 | 1.037 |
| Intend to download the app | 4 | 1 | 3.37 | 1.135 |
| **The app is useful to me personally ^a c^** |  |  |  |  |
| Overall sample statistic | 4 | 1 | 3.75 | 0.974 |
| Downloaded and still have the app | 4 | 2 | 3.84 | 0.944 |
| Downloaded and deleted the app | 3 | 2 | 3.19 | 1.174 |
| Intend to download the app | 4 | 1 | 3.72 | 0.822 |
| **The app is useful to wider society ^a c^** |  |  |  |  |
| Overall sample statistic | 4 | 1 | 4.02 | 0.903 |
| Downloaded and still have the app | 4 | 1 | 4.11 | 0.874 |
| Downloaded and deleted the app | 4 | 1 | 3.47 | 1.096 |
| Intend to download the app | 4 | 1 | 4.04 | 0.747 |
| **It is important to me that I can get an explanation for any information given to me by the app** |  |  |  |  |
| Overall sample statistic | 4 | 1 | 3.98 | 0.848 |
| Downloaded and still have the app | 4 | 1 | 4.01 | 0.801 |
| Downloaded and deleted the app | 4 | 2 | 3.72 | 1.028 |
| Intend to download the app | 4 | 1 | 4.01 | 0.874 |
| **It is important to me that I can verify that notifications from the app are authentic ^a c^** |  |  |  |  |
| Overall sample statistic | 4 | 1 | 4.08 | 0.887 |
| Downloaded and still have the app | 4 | 1 | 4.14 | 0.823 |
| Downloaded and deleted the app | 4 | 1 | 3.60 | 1.137 |
| Intend to download the app | 4 | 1 | 4.14 | 0.858 |
| **The regulations governing the creation of the app are sufficient ^a b^** |  |  |  |  |
| Overall sample statistic | 4 | 1 | 3.64 | 0.942 |
| Downloaded and still have the app | 4 | 1 | 3.72 | 0.934 |
| Downloaded and deleted the app | 3 | 1 | 3.37 | 1.089 |
| Intend to download the app | 4 | 1 | 3.51 | 0.823 |
| **It is important to me to be able to speak to a person about any advice given by app ^b^** |  |  |  |  |
| Overall sample statistic | 4 | 1 | 3.75 | 0.972 |
| Downloaded and still have the app | 4 | 1 | 3.71 | 0.982 |
| Downloaded and deleted the app | 4 | 1 | 3.64 | 1.099 |
| Intend to download the app | 4 | 1 | 4.00 | 0.804 |
| **It is important to me that I can opt-in and opt-out of contact tracing ^b^** |  |  |  |  |
| Overall sample statistic | 4 | 1 | 3.57 | 1.111 |
| Downloaded and still have the app | 4 | 1 | 3.50 | 1.166 |
| Downloaded and deleted the app | 4 | 1 | 3.58 | 0.947 |
| Intend to download the app | 4 | 1 | 3.81 | 0.954 |
| **The app is easy to use (n=571) ^a^** |  |  |  |  |
| Overall sample statistic | 4 | 1 | 4.07 | 0.897 |
| Downloaded and still have the app | 4 | 1 | 4.18 | 0.815 |
| Downloaded and deleted the app | 4 | 1 | 3.42 | 1.082 |
| **I felt that I had no choice but to download the app (n=571)** |  |  |  |  |
| Overall sample statistic | 3 | 2 | 3.02 | 1.261 |
| Downloaded and still have the app | 3 | 2 | 2.98 | 1.273 |
| Downloaded and deleted the app | 3 | 2 | 3.27 | 1.162 |
| **I have felt frustrated as a result of a notification from the app (n=571) ^a^** |  |  |  |  |
| Overall sample statistic | 3 | 2 | 2.69 | 1.284 |
| Downloaded and still have the app | 2 | 2 | 2.59 | 1.279 |
| Downloaded and deleted the app | 3 | 2 | 3.28 | 1.154 |

**Table S3. Levels of agreement with statements related to trust in the NHS Covid-19 app, n=1001**

Independent samples Kruskall-Wallis test with post-hoc examination using the Bonferroni correction, significance level *P*<.05, shows significant differences indicated by:

^a^ between Downloaded and still have the app and Do not intend to download the app

^b^ between Downloaded and still have the app and Downloaded and deleted the app

^c^ between Downloaded and still have the app and Intend to download the app

^d^ between Do not intend to download the app and Downloaded and deleted the app

^e^ between Do not intend to download the app and Intend to download the app

^f^ between Downloaded and deleted the app and Intend to download the app

|  | **Median** | **IQR** | **Mean** | **SD** |
| --- | --- | --- | --- | --- |
| **I trust that the data collected by app is used responsibly ^a b d e f^** |  |  |  |  |
| Overall sample statistic | 4 | 1 | 3.49 | 1.171 |
| Downloaded and still have the app | 4 | 1 | 3.98 | 0.888 |
| Do not intend to download the app | 3 | 2 | 2.63 | 1.224 |
| Downloaded and deleted the app | 3 | 2 | 3.23 | 1.121 |
| Intend to download the app | 4 | 1 | 3.75 | 0.842 |
| **I trust that the data collected by the app is stored securely ^a b d e f^** |  |  |  |  |
| Overall sample statistic | 4 | 1 | 3.48 | 1.161 |
| Downloaded and still have the app | 4 | 2 | 3.93 | 0.916 |
| Do not intend to download the app | 3 | 1 | 2.56 | 1.149 |
| Downloaded and deleted the app | 3 | 1 | 3.35 | 1.109 |
| Intend to download the app | 4 | 1 | 3.82 | 0.855 |
| **I feel that the app is reliable ^a b c d e f^** |  |  |  |  |
| Overall sample statistic | 4 | 1 | 3.37 | 1.162 |
| Downloaded and still have the app | 4 | 2 | 3.89 | 0.919 |
| Do not intend to download the app | 2 | 1 | 2.42 | 1.062 |
| Downloaded and deleted the app | 3 | 2 | 3.14 | 1.159 |
| Intend to download the app | 4 | 1 | 3.63 | 0.831 |
| **I trust that the app will do what it is supposed to do ^a b d e f^** |  |  |  |  |
| Overall sample statistic | 4 | 1 | 3.49 | 1.171 |
| Downloaded and still have the app | 4 | 1 | 3.97 | 0.880 |
| Do not intend to download the app | 3 | 1 | 2.53 | 1.136 |
| Downloaded and deleted the app | 3 | 2 | 3.15 | 1.216 |
| Intend to download the app | 4 | 0 | 3.95 | 0.751 |
| **I think the NHS Covid-19 app is basically trustworthy ^a b d e f^** |  |  |  |  |
| Overall sample statistic | 4 | 1 | 3.56 | 1.118 |
| Downloaded and still have the app | 4 | 1 | 4.03 | 0.875 |
| Do not intend to download the app | 3 | 1 | 2.73 | 1.129 |
| Downloaded and deleted the app | 3 | 1 | 3.21 | 0.996 |
| Intend to download the app | 4 | 1 | 3.85 | 0.879 |
| **I think that most other people will download the app ^a b e f^** |  |  |  |  |
| Overall sample statistic | 3 | 2 | 3.26 | 1.114 |
| Downloaded and still have the app | 4 | 1 | 3.58 | 1.026 |
| Do not intend to download the app | 3 | 1 | 2.65 | 1.086 |
| Downloaded and deleted the app | 3 | 2 | 3.01 | 1.055 |
| Intend to download the app | 4 | 1 | 3.48 | 0.913 |
| **I trust that most other people will self-isolate if told to do so by the app ^a d e^** |  |  |  |  |
| Overall sample statistic | 3 | 2 | 3.29 | 1.146 |
| Downloaded and still have the app | 4 | 1 | 3.54 | 1.113 |
| Do not intend to download the app | 3 | 2 | 2.80 | 1.106 |
| Downloaded and deleted the app | 3 | 1 | 3.22 | 1.072 |
| Intend to download the app | 4 | 1 | 3.45 | 1.056 |
| **I trust that my data will be deleted when the app says it will ^a b d e f^** |  |  |  |  |
| Overall sample statistic | 4 | 1 | 3.42 | 1.187 |
| Downloaded and still have the app | 4 | 2 | 3.93 | 0.892 |
| Do not intend to download the app | 2 | 2 | 2.48 | 1.196 |
| Downloaded and deleted the app | 3 | 2 | 3.09 | 1.120 |
| Intend to download the app | 4 | 1 | 3.75 | 0.851 |
| **It is important to me that I trust the app in order to use it ^a d e^** |  |  |  |  |
| Overall sample statistic | 4 | 1 | 4.01 | 0.914 |
| Downloaded and still have the app | 4 | 1 | 4.15 | 0.736 |
| Do not intend to download the app | 4 | 2 | 3.86 | 1.145 |
| Downloaded and deleted the app | 4 | 1 | 3.63 | 0.980 |
| Intend to download the app | 4 | 1 | 4.11 | 0.723 |

**Table S4. Levels of trust in groups involved in the Test and Trace system, n=1001**

Independent samples Kruskall-Wallis test with post-hoc examination using the Bonferroni correction, significance level *P*<.05, shows significant differences indicated by:

^a^ between Downloaded and still have the app and Do not intend to download the app

^b^ between Downloaded and still have the app and Downloaded and deleted the app

^c^ between Downloaded and still have the app and Intend to download the app

^d^ between Do not intend to download the app and Downloaded and deleted the app

^e^ between Do not intend to download the app and Intend to download the app

^f^ between Downloaded and deleted the app and Intend to download the app

|  | **Median** | **IQR** | **Mean** | **SD** |
| --- | --- | --- | --- | --- |
| **The big tech companies, such as Google and Apple ^a d e^** |  |  |  |  |
| Overall sample statistic | 3 | 2 | 3.18 | 1.107 |
| Downloaded and still have the app | 4 | 1 | 3.42 | 0.996 |
| Do not intend to download the app | 3 | 1 | 2.65 | 1.134 |
| Downloaded and deleted the app | 3 | 2 | 3.09 | 1.217 |
| Intend to download the app | 3 | 1 | 3.45 | 0.970 |
| **Private contractors, such as Serco ^a d e^** |  |  |  |  |
| Overall sample statistic | 3 | 2 | 2.83 | 1.103 |
| Downloaded and still have the app | 3 | 2 | 3.07 | 1.086 |
| Do not intend to download the app | 2 | 2 | 2.29 | 1.021 |
| Downloaded and deleted the app | 3 | 2 | 3.02 | 1.095 |
| Intend to download the app | 3 | 2 | 2.95 | 0.942 |
| **Small hospitality venues, such as independent pubs and cafes ^a^** |  |  |  |  |
| Overall sample statistic | 4 | 1 | 3.44 | 0.988 |
| Downloaded and still have the app | 4 | 1 | 3.62 | 0.864 |
| Do not intend to download the app | 3 | 2 | 3.13 | 1.125 |
| Downloaded and deleted the app | 4 | 1 | 3.47 | 1.085 |
| Intend to download the app | 4 | 1 | 3.45 | 0.868 |
| **Larger hospitality venues, such as chain restaurants ^a d e^** |  |  |  |  |
| Overall sample statistic | 3 | 1 | 3.32 | 1.007 |
| Downloaded and still have the app | 4 | 1 | 3.52 | 0.923 |
| Do not intend to download the app | 3 | 2 | 2.91 | 1.068 |
| Downloaded and deleted the app | 4 | 1 | 3.43 | 1.048 |
| Intend to download the app | 3 | 1 | 3.41 | 0.818 |
| **The UK Government ^a b e^** |  |  |  |  |
| Overall sample statistic | 3 | 2 | 3.01 | 1.278 |
| Downloaded and still have the app | 4 | 2 | 3.32 | 1.213 |
| Do not intend to download the app | 2 | 2 | 2.39 | 1.181 |
| Downloaded and deleted the app | 3 | 2 | 2.80 | 1.298 |
| Intend to download the app | 3 | 2 | 3.27 | 1.207 |
| **My local council ^a d e^** |  |  |  |  |
| Overall sample statistic | 3 | 1 | 3.30 | 1.059 |
| Downloaded and still have the app | 4 | 1 | 3.51 | 0.968 |
| Do not intend to download the app | 3 | 2 | 2.86 | 1.096 |
| Downloaded and deleted the app | 4 | 2 | 3.35 | 1.153 |
| Intend to download the app | 4 | 1 | 3.49 | 0.905 |
| **The NHS ^a b e f^** |  |  |  |  |
| Overall sample statistic | 4 | 1 | 4.11 | 0.946 |
| Downloaded and still have the app | 4 | 1 | 4.33 | 0.774 |
| Do not intend to download the app | 4 | 2 | 3.72 | 1.102 |
| Downloaded and deleted the app | 4 | 1 | 3.81 | 0.989 |
| Intend to download the app | 4 | 1 | 4.32 | 0.783 |
